# Inhibitory effect of saliva on osteoclastogenesis in vitro requires toll-like receptor 4 signaling

**DOI:** 10.1007/s00784-016-2041-7

**Published:** 2017-01-18

**Authors:** Heinz-Dieter Müller, Jordi Caballé-Serrano, Adrian Lussi, Reinhard Gruber

**Affiliations:** 1 Department of Preventive, Restorative and Pediatric Dentistry, School of Dental Medicine, University of Bern, Freiburgstrasse 7, 3010 Bern, Switzerland; 2 Department of Oral Surgery and Stomatology, School of Dental Medicine|, University of Bern, Bern, Switzerland; 3 Department of Oral and Maxillofacial Surgery, School of Dental Medicine, Universitat Internacional de Catalunya, Barcelona, Spain; 4 Department of Oral Biology, Medical University of Vienna, Sensengasse 2a, 1090 Vienna, Austria

**Keywords:** Saliva, Toll-like receptor, Endotoxin, Osteoclast, Murine bone marrow, Dentistry

## Abstract

**Objectives:**

Saliva can suppress osteoclastogenesis, but the underlying mechanism has not been discovered yet. Considering that endotoxins suppress osteoclastogenesis in bone marrow cultures and that saliva contains endotoxins, it was reasonable to hypothesize that the impact of saliva on osteoclastogenesis requires toll-like receptor 4 signaling.

**Material and methods:**

To test this hypothesis, we blocked toll-like receptor 4 signaling with TAK-242 in the presence of saliva in murine bone marrow cultures. Osteoclastogenesis was evaluated based on gene expression analysis and histochemical staining for tartrate-resistant acid phosphatase. Resorption was performed on dentine.

**Results:**

We report that TAK-242 reversed the inhibitory effect of fresh sterile saliva on the formation of multinucleated cells that stained positive for tartrate-resistant acid phosphatase. In line with this finding, TAK-242 increased the expression of the osteoclast functional genes cathepsin K, calcitonin receptor, and tartrate-resistant acid phosphatase in the presence of saliva. TAK-242 also supported the expression of NFATc1, the master regulator of osteoclastogenesis, as well as DC-STAMP and Atp6v0d2, both being cell fusion genes. In support of the hypothesis, depletion of saliva from endotoxin partially reversed the inhibitory effect on osteoclastogenesis. Moreover, salivary pellicle on plastic and titanium did not affect osteoclastogenesis.

**Conclusion:**

Inhibition of toll-like receptor 4 signaling revealed that saliva can contribute to innate immunity by preventing hematopoietic progenitors to become osteoclasts.

**Clinical relevance:**

Saliva can activate pattern recognition receptor signaling through endotoxins and other stress factors, indicating the demand for macrophages rather than for osteoclasts.

**Electronic supplementary material:**

The online version of this article (doi:10.1007/s00784-016-2041-7) contains supplementary material, which is available to authorized users.

## Introduction

Saliva is a complex pleiotropic biological fluid produced by the salivary glands. The broad range of physiological functions covers lubrication of the oral mucosa, enzymatic food digestion [1], and formation of the pellicle layer [2]. Besides the molecules produced by the glands, saliva also contains products of the commensalistic microbiota, making this biological fluid even more complex in its composition [3]. Thus, saliva has traditionally been used for diagnostic purposes in oral pathology [4, 5] and in general medicine [4]. Diagnostic assays have advanced from single analyst to screening approaches, including proteomics [6] and microbiomics [3]. What remains to be advanced are functional assays describing the cellular response to saliva and its components. Saliva reaches all defect sites in the oral cavity, including the alveolar bone after tooth extraction. Particularly, in vivo research with desalivated rats showed a delay in socket healing and slower bone remodeling [7, 8]. The molecular mechanism on how saliva impacts bone in the oral microenvironment is unclear.

Functional assays revealed that saliva is a powerful inhibitor of osteoclastogenesis, while allowing the formation of phagocytes in murine bone marrow cultures to occur [9]. The underlying molecular mechanisms are unknown. Saliva also provokes a strong inflammatory response in oral fibroblasts [10, 11], which has recently been attributed to toll-like receptor (TLR) 4 signaling [12]. This discovery was based on TAK-242, a potent small-molecule-specific inhibitor of TLR4 signaling that was originally developed to cope with sepsis [13]. Mechanistically, TAK-242 prevents the association of TLR4 with adaptor proteins [14]. Saliva contains endotoxins of gram-negative bacteria such as lipopolysaccharides that activate TLR4. TAK-242 can thus be used to investigate the role of TLR4 signaling to mediate the inhibitor effect of saliva on osteoclastogenesis. Support for this assumption comes from in vitro studies showing that lipopolysaccharides alone are potent inhibitors of osteoclastogenesis in bone marrow cultures [15, 16].

Osteoclastogenesis in bone marrow cultures can be induced by the addition of two molecules, RANKL initiating the differentiation process and M-CSF promoting the survival and expansion of the respective myeloid progenitor cells [17]. TGF-β supports this process further [18]. Differentiation requires signaling via RANK [19] and the associated factor TRAF6 [20]. The pathway increases the expression of the nuclear factor of activated T cells c1 (NFATc1), the master regulators of osteoclastogenesis [21]. Osteoclast fusion requires dendritic cell-specific transmembrane protein (DC-STAMP) and ATPase, H^+^ transporting, lysosomal 38 kDa, and V0 subunit d2 (Atp6v0d2) [22, 23], which is also regulated by NFATc1 at the transcriptional level.

Co-stimulatory molecules contribute to osteoclastogenesis by activating the immunoreceptor tyrosine-based activation motif (ITAM)-dependent pathway [24]. Osteoclast-associated receptor (OSCAR) and triggering receptor expressed in myeloid cells (TREM2) are receptors that are associated with the adaptor molecules Fc receptor common gamma chain (FcRγ) and DNAX-activating protein 12 kDa (DAP12), respectively. Finally, osteoclasts are characterized by multinuclearity and the expression of functional genes, including tartrate-resistant acid phosphatase (TRAP), cathepsin K (CatK), and the calcitonin receptor (CTR). The expression of the respective genes provides insight into the process of osteoclastogenesis in vitro. We show here that TAK-242 reversed the inhibitory effect of fresh sterile saliva on the formation of osteoclasts.

## Materials and methods

### Saliva sampling and treatment

Human whole saliva was collected from the group of authors who had no oral inflammation and were non-smokers, as recently reported [9–11, 25]. Saliva flow was stimulated chewing paraffin wax (Ivoclar Vivadent AG, Schaan, Liechtenstein) and collected between 09:00 and 11:00 a.m. Saliva was centrifuged at 4000×*g* for 5 min, and filtered (0.22 μm PES syringe filter, TPP AG, Trasadingen, Switzerland) samples were used. For preparing a saliva pellicle [25], culture plates and titanium disks (grade 4 titanium, machined surface; Institut Straumann AG, Basel, Switzerland) were exposed to whole saliva for 2 h, followed by two steps of vigorous washing with phosphate-buffered saline [25].

### In vitro osteoclastogenesis in bone marrow cultures

Bone marrow cells from 4- to 6-week-old female BALB/c mice (Veterinary service, Department of Clinical Research, University of Bern) were seeded at one million bone marrow cells per square centimeter in alpha modified Eagle’s Minimum Essential Medium supplemented with 10% fetal calf serum (FCS) and antibiotics after approval of the Ethics Committee (No. BE76/12) of the University of Bern. Receptor activator of nuclear factor kappa-B ligand (RANKL, 30 ng/ml), macrophage colony-stimulating factor (M-CSF, 30 ng/ml), and human transforming growth factor beta1 (TGF-β1, 10 ng/ml) were used to induce osteoclastogenesis. All factors were obtained from ProSpec (Ness-Ziona, Israel). If not otherwise indicated, 10% saliva was included in the culture medium. Pharmacological blocking was performed with 25 μM of TAK-242 (Merck Millipore, Darmstadt, Germany). Endotoxin removal resins were used to deplete saliva from lipopolysaccharides (EndoTrap HD, Hyglos, Bernried, Germany). In indicated experiments, bone marrow cells were exposed to 10 μg/ml lipopolysaccharides (LPS) with or without TAK-242 (25 μM). After 5 days, histochemical staining for tartrate-resistant acid phosphatase (TRAP, Sigma-Aldrich) was performed. Cells with three or more nuclei were counted positive for osteoclasts. For resorption assays, bone marrow cells were seeded onto dentine slices for 5 days, with or without saliva and TAK-242. Prior to cell seeding, dentine disks were cleaned with ultra-sonication treatment and sterilized by UV light exposure. After 5 days, cells were detached with sodium hypochlorite (10 min) and ultra-sonication (30 min). Resorption lacunae were imaged via scanning electron microscopy at 100-fold magnification (JSM-6010PLUS/LA, Jeol, Japan) (Table 1).

**Table 1:** Relative gene expression of osteoclast-like cells exposed to autoclaved saliva

Gene	autoclaved saliva (121 °C)	SD	Autoclaved saliva (121 °C) + TAK-242 (25 μM)	SD
CatK	0.01*	0.01	1.16	0.23
TRAP	0.01*	0.01	0.71	0.03
CTR	0.00*	0.00	0.28	0.10

Osteoclast-like cells were exposed to heated sterile saliva (121 °C), with or without 25 μM of TLR4-receptor inhibitor TAK-242. Data represent the gene expression relative to the untreated control

*p values <0.05

### Expression of marker genes in bone marrow cultures

Total RNA was isolated using the High Pure RNA Isolation Kit (Roche Applied Science, Rotkreuz, Switzerland). Reverse transcription (RT) was performed with Transcriptor Universal cDNA Master, and PCR was performed with TaqMan Universal PCR Master Mix (Applied Biosystems, Carlsbad, CA, USA) or the FastStart Universal Probe Master Rox on a 7500 Real-Time PCR System (Roche). Probes for CTR, TRAP, CatK, OSCAR, TREM2, FcRγ, DAP12, and beta actin were obtained from the TaqMan Gene Expression Assays service (Applied Biosystems). In all experiments, the FastStart Universal SYBR Green Master Rox (Roche) was used. All other primers were designed with the online Universal ProbeLibrary System (Table 2) [9]. The messenger RNA (mRNA) levels were calculated by normalizing to the housekeeping gene beta actin using the ΔΔCt method.

**Table 2:** Primer sequences of the investigated genes

Gene	Forward primer	Reverse primer	Reference
m βactin	ctaaggccaaccgtgaaaag	accagaggcatacagggaca	[9]
mRANK	gtgctgctcgttccactg	agatgctcataatgcctctcct	[9]
m c-fos	gcaactttctatgacactgaaacac	tctctctagggctgcattgg	[9]
mNFATc-1	ccgttgcttccagaaaataaca	tgtgggatgtgaactcggaa	[9]
mDC-Stamp	aagctccttgagaaacgatca	caggactggaaaccagaaatg	[9]
mAtp6Od2	aagcctttgtttgacgctgt	gccagcacattcatctgtacc	[9]
mTRAF6	ttgcacattcagtgtttttgg	tgcaagtgtcgtgccaag	[9]
mCXLC2	aaaatcatccaaaagatactgaacaa	ctttggttcttccgttgagg	[9]
mCCL2	catccacgtgttggctca	gatcatcttgctggtgaatgagt	[9]

### Cell viability and proliferation

Bone marrow cells were stimulated with the selected preparations for 5 days and subjected to viability or proliferation assays. The viability measures were determined via formazan formation assay (Sigma, St. Louis, USA), Live-Dead Staining Kit from Enzo Life Sciences AG (Lausen, Switzerland), and the DNA incorporation of 5-Bromo-2´-Deoxyuridine (BrdU) Cell Proliferation ELISA Kit (Roche Life Science, Penzberg, Germany).

### Statistical analysis

Data were compared using ANOVA and Student’s *t* test. For post hoc analysis, the *p* value was adjusted according to the Tukey’s test. At least three different experiments with two donors were performed if not indicated otherwise. Statistical analysis was performed using GraphPad Prism 6.0 (GraphPad Software Inc., San Diego, USA), *p* < 0.05.

## Results

### TAK-242 reversed the inhibitory effect of sterile saliva on osteoclastogenesis

**Fig. 1:**
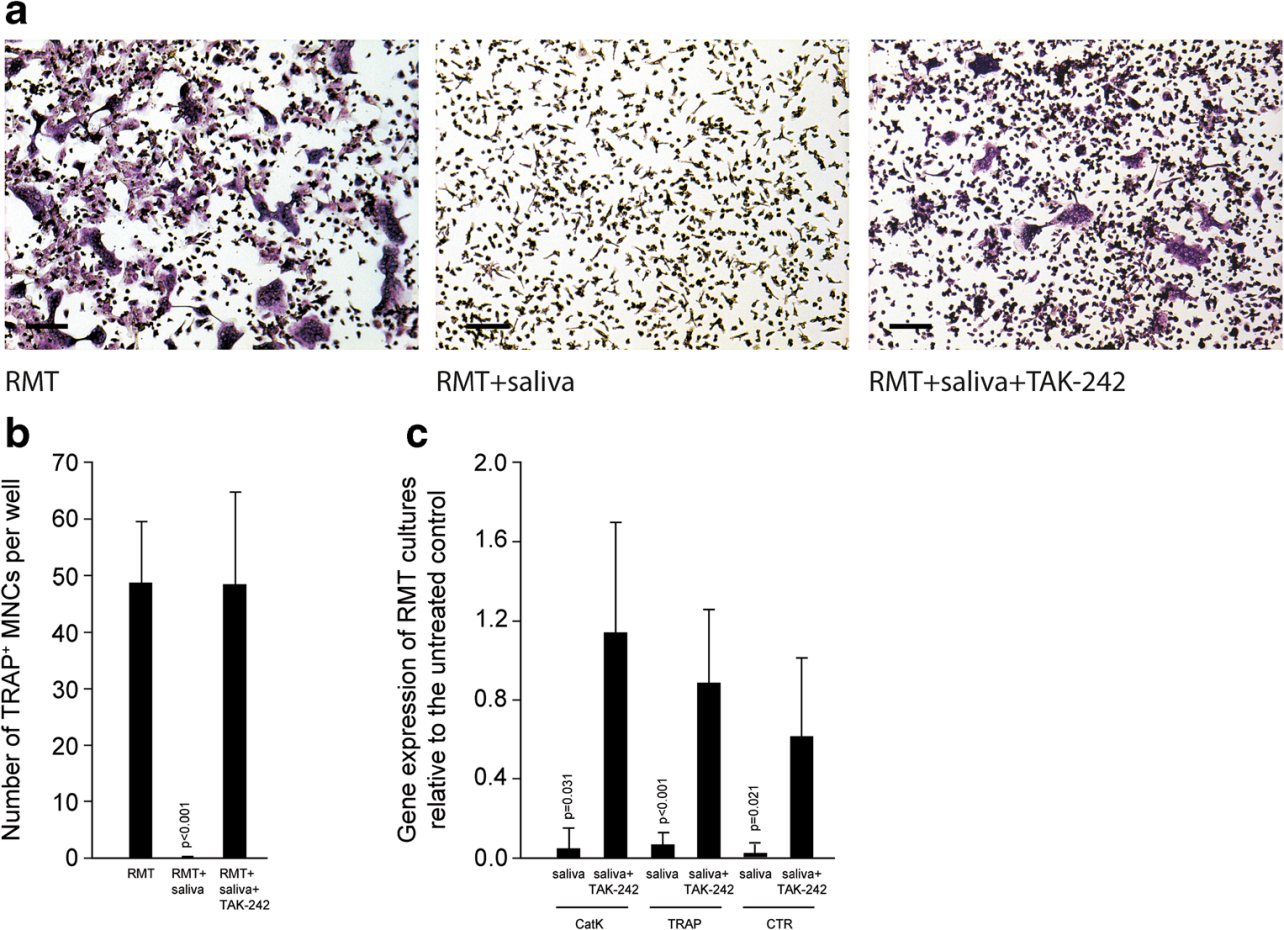
TAK-242 reversed the inhibitory effect of sterile saliva on osteoclastogenesis. Bone marrow cells from mice were grown with and without the presence of the TLR4 inhibitor TAK-242, in the presence of an osteoclastogenesis inducer cocktail consisting of RANKL, M-CSF, and TGF-β (RMT). Osteoclastogenesis is indicated by histochemical staining of TRAP in multinucleated cells. *Bars* represent 100 μm. TAK-242 greatly reversed the inhibitory effect of saliva on osteoclastogenesis (**a**, **b**). In support of the histological picture, also the expression of osteoclast functional genes CatK, TRAP, and CTR was increased by TAK-242, even reaching the levels of controls with no saliva (**c**). Data were normalized to positive expression levels of RMT cultures. *Bars* represent the mean ± standard deviation of in total five experiments. Not indicated are *p* values >0.1

**Fig. 2:**
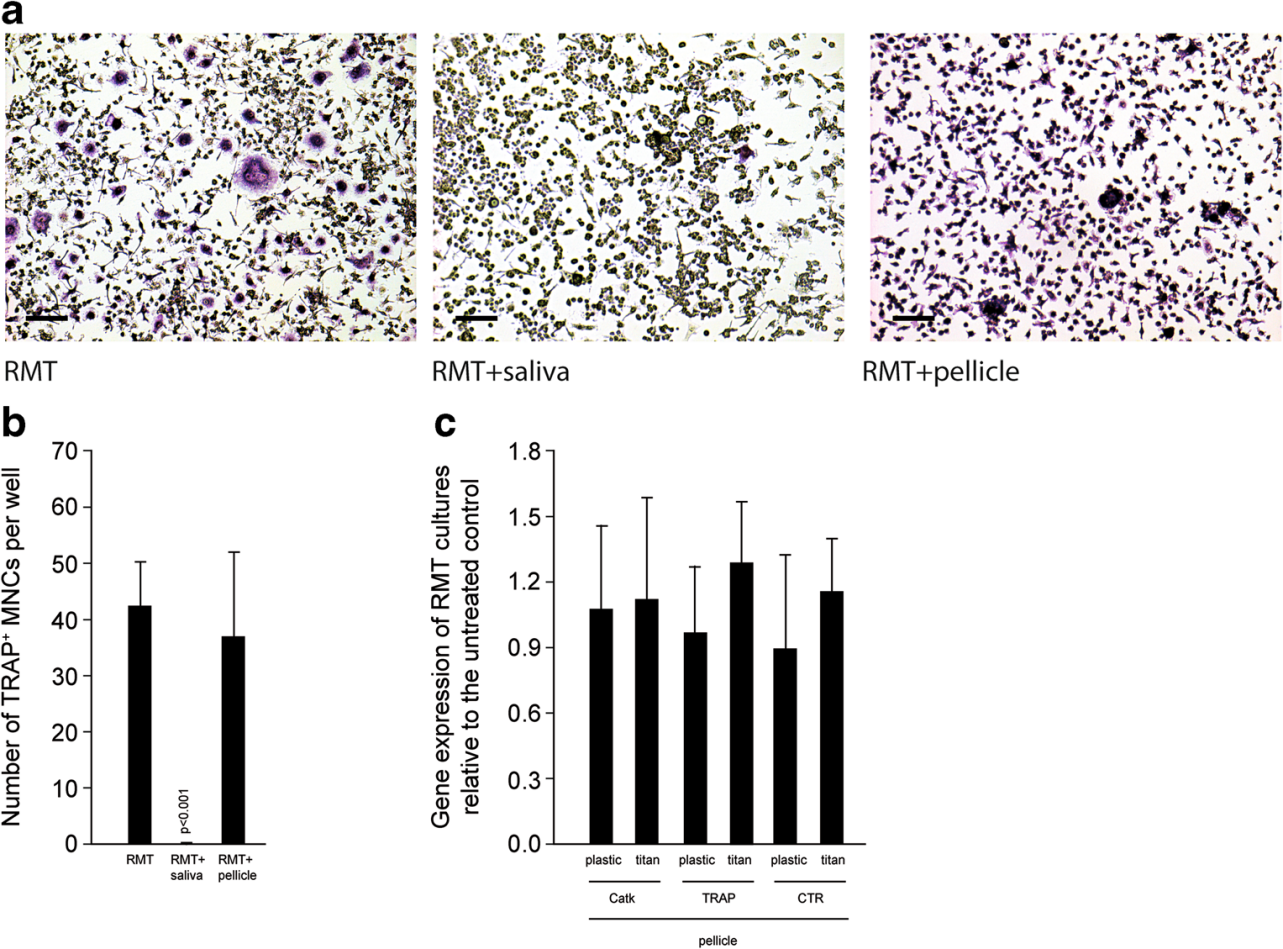
Saliva pellicle does not affect osteoclastogenesis. Murine bone marrow cultures were grown onto salivary pellicle on plastic and titanium with an osteoclastogenesis inducer cocktail consisting of RANKL, M-CSF, and TGF-β (RMT). Salivary pellicle did not affect formation of multinucleated TRAP^+^ cells on plastic (**a**, **b**). Expression of osteoclast functional genes CatK, TRAP, and CTR was not affected by salivary pellicle (**a**). Data were normalized to positive expression levels of RMT cultures. *Bars* represent the mean ± standard deviation of in total five experiments. Not indicated are *p* values >0.1

To examine the influence of endotoxins within saliva on osteoclastogenesis, murine bone marrow cells were grown in the presence of TAK-242, besides the factors RANKL, M-CSF, and TGF-β. As reported recently, saliva is a potent suppressor of osteoclastogenesis, indicated by the formation of TRAP-positive multinucleated cells [9]. Importantly, TAK-242 reversed the inhibitory effect of sterile saliva and LPS on osteoclastogenesis, shown by the expression of osteoclast genes Catk, TRAP, and CTR. Thus, blocking TLR4 signaling with TAK-242 allowed osteoclastogenesis in the presence of saliva (Fig. 1) and LPS (Suppl. Fig. 1). Salivary pellicle on plastic and titanium did not affect the formation of TRAP-positive multinucleated cells (Fig. 2a, b) and the expression of osteoclast genes CatK, TRAP, and CTR (Fig. 2c), even though the pellicle delayed adhesion of cells within the first 24 h (data not shown).

### TAK-242 blocked the effect of saliva on the master regulator of osteoclastogenesis and the fusion genes

**Fig. 3:**
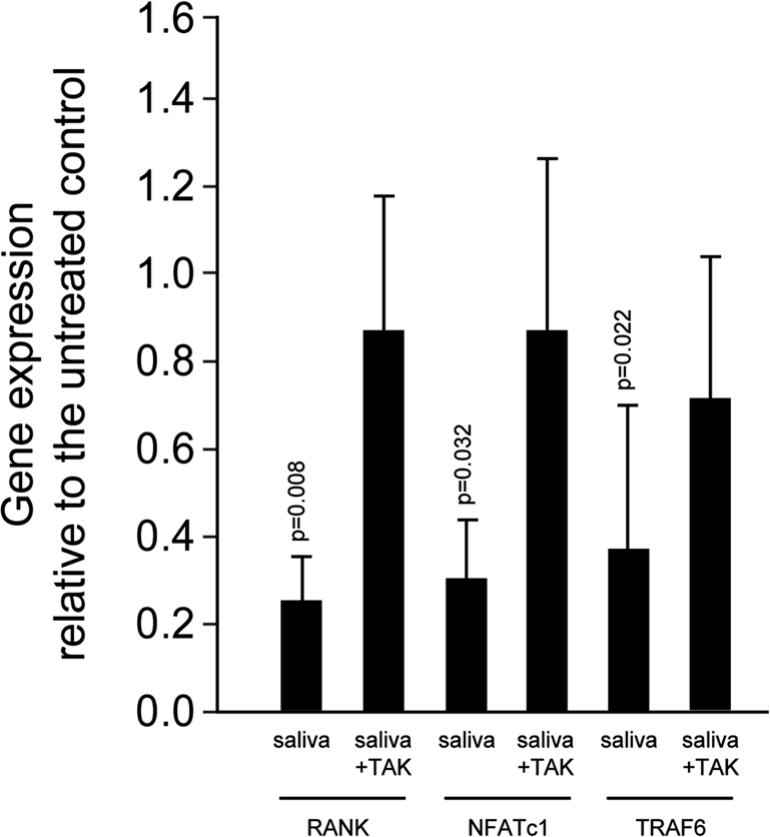
TAK-242 blocked the effect of saliva on the master regulator of osteoclastogenesis and the fusion genes. When bone marrow cells were grown in the presence of TAK-242, the suppressed expression of the signaling molecules RANK, TRAF6, and NFATC1 was reversed, reaching levels of control cultures without saliva. Data were normalized to positive expression levels of RMT cultures. *Bars* represent the mean ± deviation of in total five experiments. Not indicated are *p* values >0.1

**Fig. 4:**
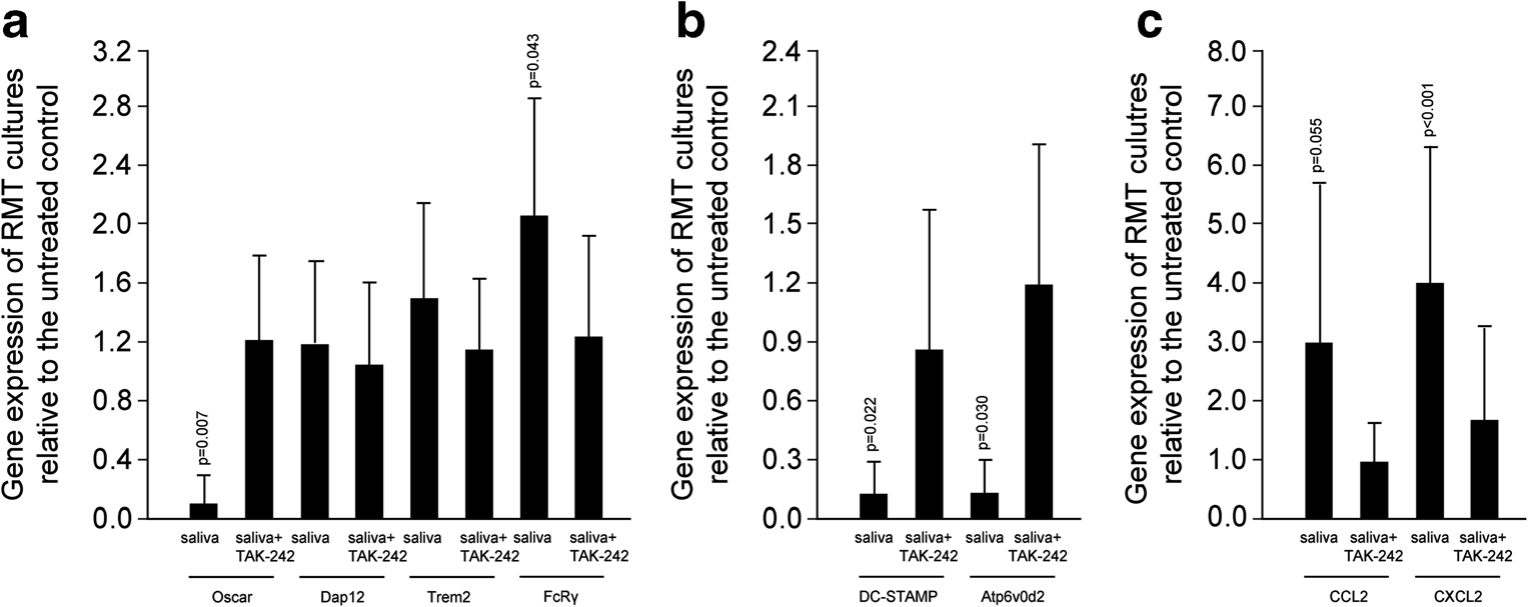
TAK-242 also reversed the inhibitory and stimulatory effect of saliva on expression of OSCAR and FcRg, respectively (**a**). Moreover, TAK-242 increased DC-STAMP and Atp6v0d2 expression, which are markedly decreased by saliva (**b**). Accordingly, the increased mRNA expression of CXCL2 and CCL2 in response to saliva was blocked by TAK-242 (**c**). Data were normalized to positive expression levels of RMT cultures. *Bars* represent the mean ± standard deviation of in total five experiments. Not indicated are *p* values >0.1

To investigate the impact of blocking TLR4 signaling on downstream mechanisms, TAK-242 was tested for changing gene expression of the RANK–RANKL signaling pathway. According to the overall hypothesis, TAK-242 competed with saliva for the expression of RANK, TRAF6, and the respective downstream master regulator NFATc1 (Fig. 3). TAK-242 also reversed the inhibitory and stimulatory effect of saliva on expression of OSCAR and FcRg, respectively (Fig. 4a). Moreover, TAK-242 increased DC-STAMP and Atp6v0d2 expression, which was markedly decreased by saliva (Fig. 4b). Accordingly, the increased mRNA expression of CXCL2 and CCL2 in response to saliva was blocked by TAK-242 (Fig. 4c). In addition, resorption assays revealed that TAK-242 canceled the inhibitory effect of saliva to the resorption capacity of osteoclast-like cells (Fig. 7). Overall, TAK-242 blocked the inhibitory effect of saliva and LPS on osteoclastogenesis in murine bone marrow cultures.

### Endotoxin removal from saliva supports osteoclastogenesis

**Fig. 5:**
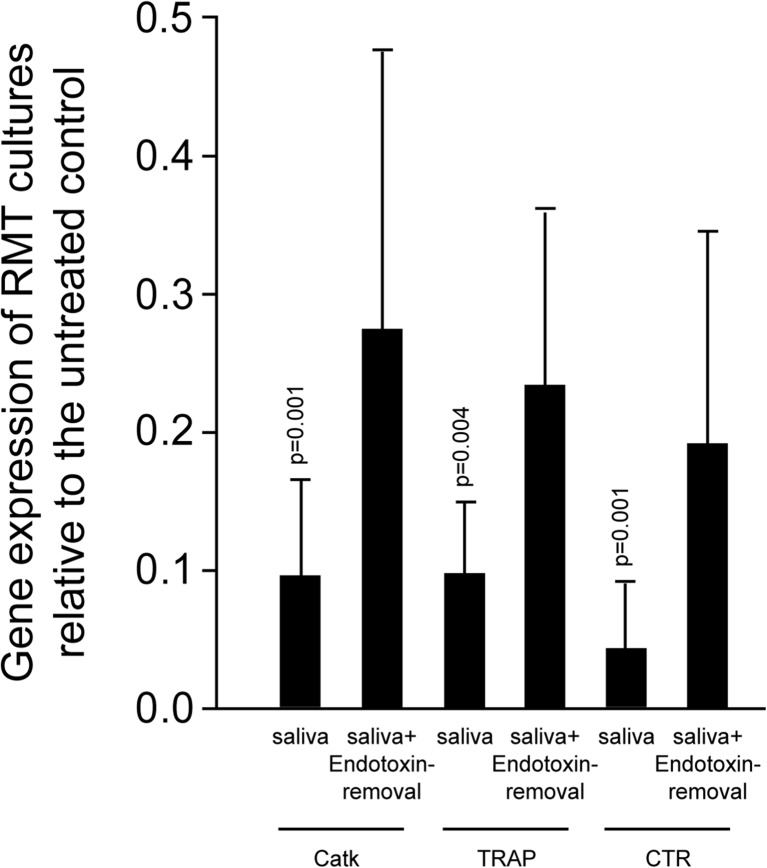
Endotoxin removal from saliva supports osteoclastogenesis. Osteoclasts were exposed to saliva or to saliva pre-treated with endotoxin removal resins. Saliva decreased Catk, TRAP, and CTR gene expression to a significant level. Saliva prior to treatment with endotoxin removal resins increased marker genes’ expression significantly compared to saliva alone. Data were normalized to positive expression levels of RMT cultures. *Bars* represent the mean ± standard deviation of in total five experiments. Not indicated are *p* values >0.1

**Fig. 6:**
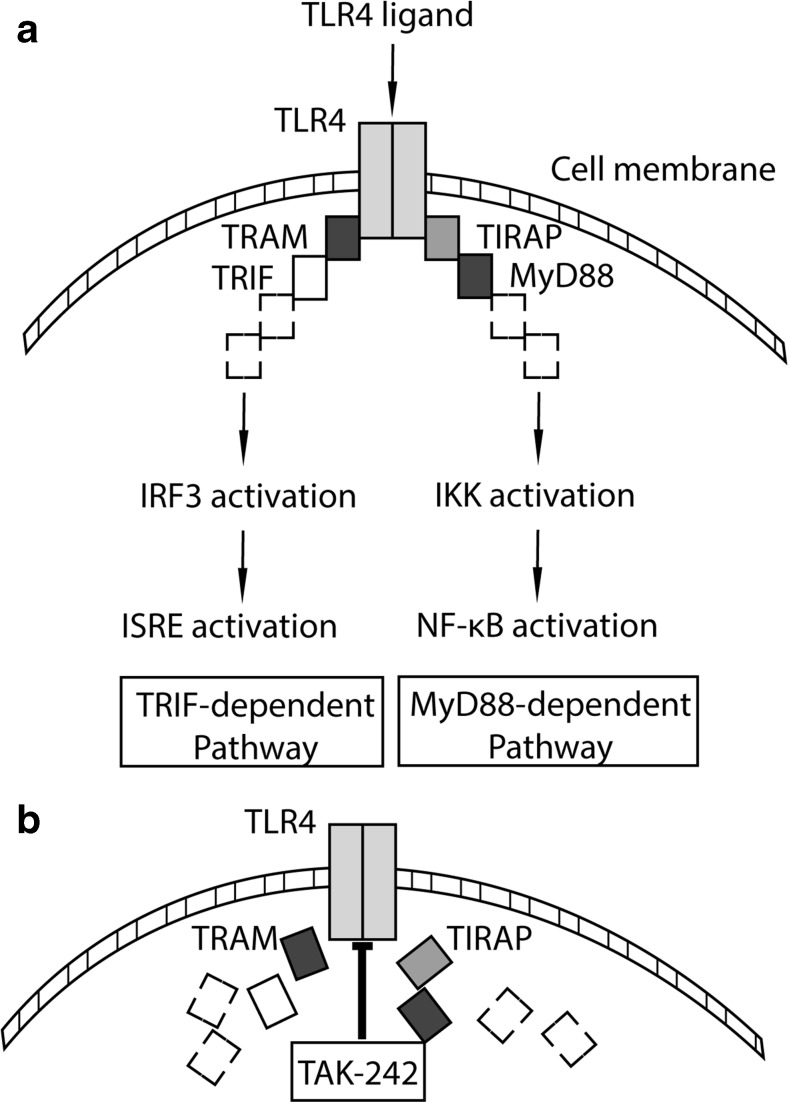
Schematic illustration of the TLR4 signaling pathway (**a**) and inhibition via TAK-242 (**b**). TLR4 signaling depends on MyD88- and TRIF-dependent signaling pathway. TAK-242 blocking the intracellular domain of TLR4. Thereby, TAK-242 interferes with interactions between TLR4 and its adaptor molecules, TIRAP and TRAM [14]

**Fig. 7:**
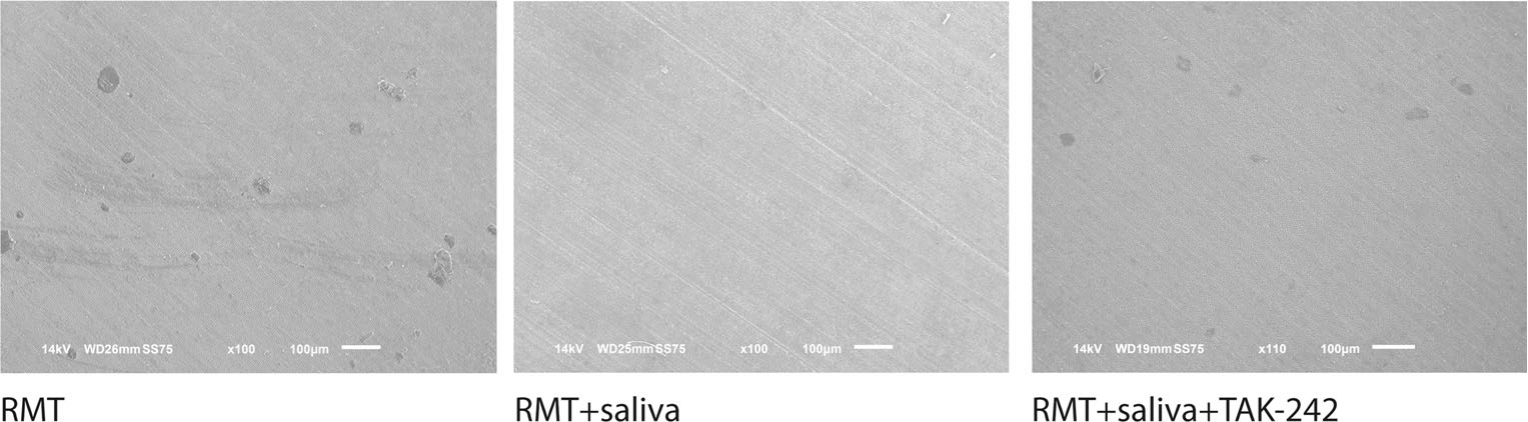
Resorption lacunae of osteoclast-like cells exposed to saliva and TAK-242. Bone marrow cells were seeded on dentin disks for 5 days in RMT medium. Cells were stimulated with or without sterile saliva and TLR4 inhibitor TAK-242. To detach the cells, dentine disks were treated with sodium hypochlorite and ultra-sonication. Resorption lacunae were imaged via scanning electron microscopy at a 100-fold magnification. Saliva exposed osteoclast-like cells showed any contribution to dentine resorption compared to saliva-exposed cells co-stimulated with TAK-242

Endotoxin removal was performed to further prove that the inhibition of osteoclastogenesis is a consequence of endotoxins in saliva. As indicated in Fig. 5, saliva being at least partially depleted from endotoxins had a less inhibitory effect on osteoclastogenesis than the respective original unprocessed saliva. Further support for the role of endotoxins comes from experiments with saliva heated up to 120 °C that was still capable to suppress osteoclastogenesis (Table 1) and LPS-exposed osteoclast experiments (Suppl. Fig. 1). Live-dead staining and proliferation assays further indicated that neither saliva nor TAK-242 causes any adverse reaction in the in vitro system (Suppl. Fig. 2). Taken together, experiments with endotoxin removal and heating of saliva point towards a role of endotoxins in mediating at least some of the effects of saliva on osteoclastogenesis. A schematic singling cascade including TAK-242 is provided in Figs. 6 and 7.

## Discussion

The major findings of the present study are that blocking in vitro osteoclastogenesis by saliva requires TLR4 signaling. Basically, the TLR4 inhibitor TAK-242 completely reversed the cellular response to saliva in vitro [9]. Further support for this hypothesis comes from observations that endotoxin removal from saliva supports osteoclastogenesis and from the resistance of the respective activity in saliva against heating. The findings are substantial because they point towards endotoxins of the commensalistic oral microbiota, thereby not ruling that also exocrine molecules of the salivary glands through TLR4 signaling target hematopoietic stem cells. The question how the hematopoietic stem cells respond to saliva at the molecular level is starting to be answered.

Saliva provokes a massive inflammatory response in oral fibroblasts [10, 11, 25]. Originally, blocking peptides raised against TLR4 and the downstream mediator MYD88 failed to reverse the inflammatory response [9], but TAK-242 was successful in this regard [12]. Saliva proteins including alpha-amylase, prolactin-inducible protein, and cystatin serve as carriers for endotoxins [26, 27], and LPS suppresses RANK expression in macrophages [28]. Also in agreement with the present findings, salivary pellicle does not suppress osteoclastogenesis and does not show a pro-inflammatory activity [25]. Our data are also in line with the fundamental research that LPS alone suppresses osteoclastogenesis in bone marrow cultures [15, 16, 29], but not in other in vitro systems with committed progenitors such as RAW 264 cells [30]. LPS even increases osteoclastogenesis when cells are committed to become osteoclasts [16]. Nevertheless, the present findings do not rule out that other, as yet undefined molecules in saliva suppress osteoclastogenesis.

The clinical relevance has to be interpreted with care, as the role of osteoclastogenesis in oral sciences has not yet been resolved. However, osteoclastogenesis might play a role after tooth extraction or in other situations where bone is exposed to saliva. For example, in vivo animal models targeting the socket healing capacity of desalivated rats show that without saliva socket healing after tooth extraction is delayed [7]. In the absence of saliva, replacement of the blood clot with granulation tissue is delayed including fewer collagen fibers and cells at the defect side [7]. Osteoclasts originate from hematopoietic progenitors that can be isolated from blood and are not restricted to the bone marrow. Thus, the murine bone marrow culture is not the exclusive bioassay for osteoclasts and does not necessarily represent the situation in a tooth extraction site. While bone marrow is a source of highly undifferentiated hematopoietic stem cells, the blood contains mainly monocytes that can become osteoclasts in vitro [31]. Thus, we cannot rule out that saliva modulates osteoclastogenesis in monocyte cultures. Nevertheless, we revealed some basic principles of TLR4 signaling activated by saliva in bone marrow cultures, observations that exceed the possible relevance of osteoclastogenesis, pointing towards a differentiation shift of hematopoietic stem cells into a macrophage lineage that is involved in the early stages of wound healing, including defects of the oral cavity. Support for this hypothesis comes from our recent research showing that saliva supports polarization of macrophages into the pro-inflammatory M1 phenotype [32].

The present findings, therefore, have to be interpreted in the sense of a functional assay using osteoclastogenesis as a read-out for understanding the biological potential of saliva and the differentiation between the molecules produced in the glands and endotoxins from the microbiota. Caution should be taken in concluding that endotoxins in saliva act as “anti-resorptive” components, considering that the bone marrow culture does not necessarily represent the clinical situation where committed progenitors appear at the defect site. In this situation, endotoxins in saliva most likely support osteoclastogenesis. Nevertheless, saliva stimulates oral wound healing [8, 33] and, considering that inflammation is part of wound healing, saliva might contribute to innate immunity by preventing macrophage progenitors to become osteoclasts, as the process involves activation of pattern recognition receptor signaling.

The limitation of the study is that the biological relevance of our findings remains to be clarified. The data, however, should encourage considering endotoxins within saliva to act as a bioactive component that is part of the physiological composition of saliva. Endotoxins in saliva might have a potential beneficial effects related to the innate immune system, which is triggered by the activation of the pattern recognition receptors—including TLR4 [34]. However, the data should not be interpreted only towards endotoxins, as saliva holds a myriad of bioactive proteins that might contribute to the TLR4-mediated cell response. It would thus be interesting to investigate the impact of saliva from sterile animals on osteoclastogenesis and, based on these preclinical models, to study the role of saliva on wound healing, including extraction sites. Future studies should therefore include more functional assays allowing the differentiation of effects of saliva components from the gland and those from the microbiom, helping to decipher the biological function of saliva in oral sciences.

## Electronic supplementary material

Suppl. Fig. 1 TAK-242 reversed the inhibitory effect of LPS on osteoclastogenesis. Bone marrow cells from mice were grown with and without the TLR4 inhibitor TAK-242 in the presence of an osteoclastogenesis inducer cocktail consisting of RANKL, M-CSF, and TGF-β (RMT). Osteoclastogenesis is indicated by histochemical staining of TRAP in multinucleated cells (a). Black bars represent 100 μm. TAK-242 greatly reversed the inhibitory effect of LPS on osteoclastogenesis (b). In support of the histological picture, also the expression of osteoclast functional genes CTR, CatK, and TRAP was increased by TAK-242 (c). Data were normalized to positive expression levels of RMT cultures. Bars represent the mean ± standard deviation of in total five experiments. Not indicated are *p* values>0.1 (GIF 454 kb)

High Resolution Image (TIFF 15216 kb)

Suppl. Fig. 2 Cell viability and proliferation in the presence of saliva, LPS and TAK-242. Osteoclast-like cells exposed to 10 μg/ml LPS showed significantly reduced viability and proliferation. For Live-dead staining, bone marrow cells were colored in green, indicating that the cells are viable (10-fold magnification) (a). The viability measures were determined via formazan formation assay (b). DNA incorporation of 5-Bromo-2´-Deoxyuridine (BrdU) Labeling and Detection Protocol was used to measure cell proliferation (c). Data were normalized to expression levels of RMT cultures. Bars represent the mean ± standard deviation of in total five experiments. Not indicated are *p* values>0.1 (GIF 198 kb)

High Resolution Image (TIFF 3689 kb)
