# Supplementary figures and images for: Inhibitory effect of saliva on osteoclastogenesis in vitro requires toll-like receptor 4 signaling
Source: Clin Oral Investig. 2017 Jan 18;21(8):2445–52. doi: 10.1007/s00784-016-2041-7 (PMC5632348; doi:10.1007/s00784-016-2041-7)

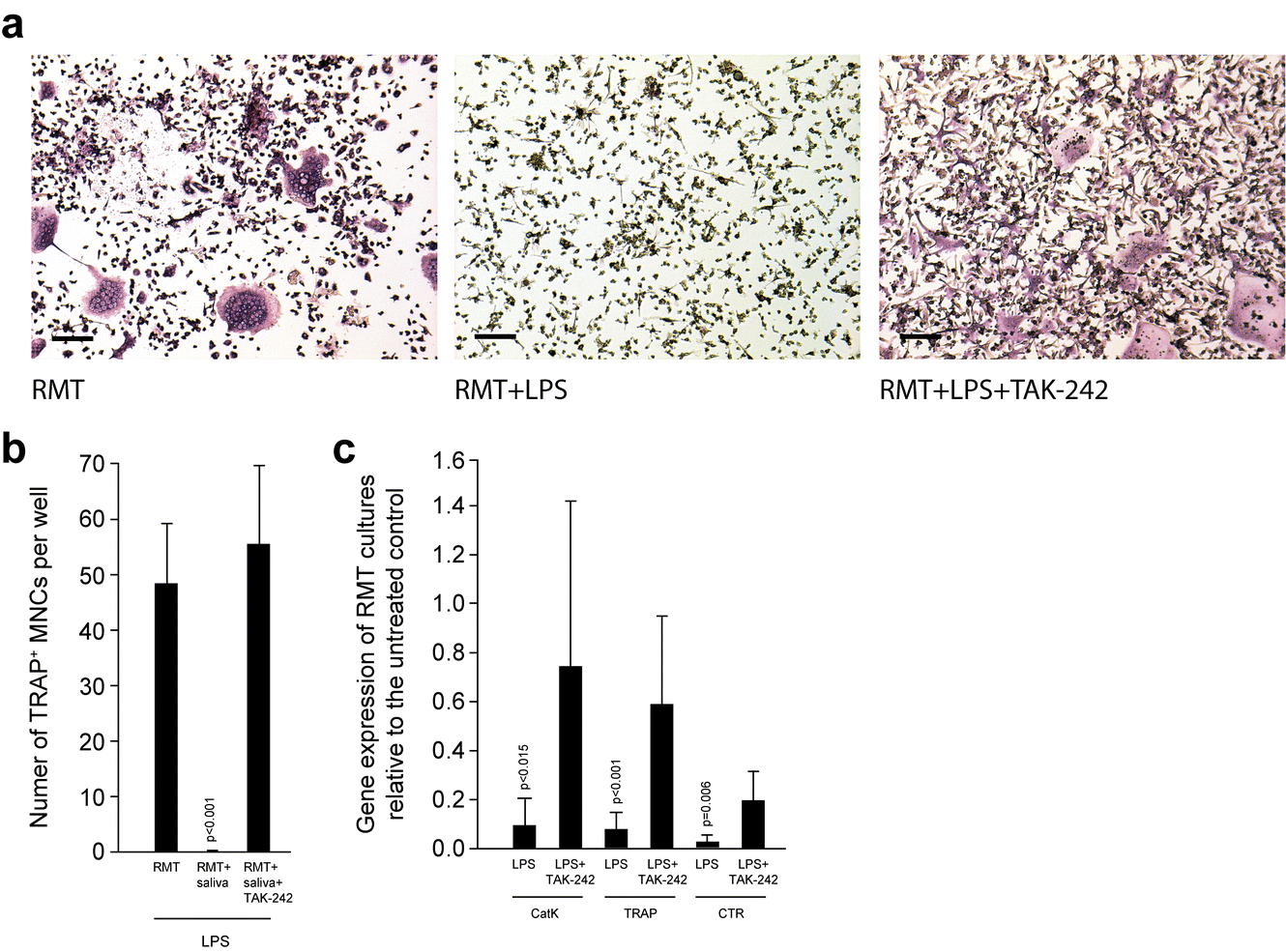

Supplement: Supplementary file 1 — TAK-242 reversed the inhibitory effect of LPS on osteoclastogenesis. Bone marrow cells from mice were grown with and without the TLR4 inhibitor TAK-242 in the presence of an osteoclastogenesis inducer cocktail consisting of RANKL, M-CSF, and TGF-β (RMT). Osteoclastogenesis is indicated by histochemical staining of TRAP in multinucleated cells (a). Black bars represent 100 μm. TAK-242 greatly reversed the inhibitory effect of LPS on osteoclastogenesis (b). In support of the histological picture, also the expression of osteoclast functional genes CTR, CatK, and TRAP was increased by TAK-242 (c). Data were normalized to positive expression levels of RMT cultures. Bars represent the mean ± standard deviation of in total five experiments. Not indicated are p values>0.1 (GIF 454 kb) [file 784_2016_2041_Fig8_ESM.gif]

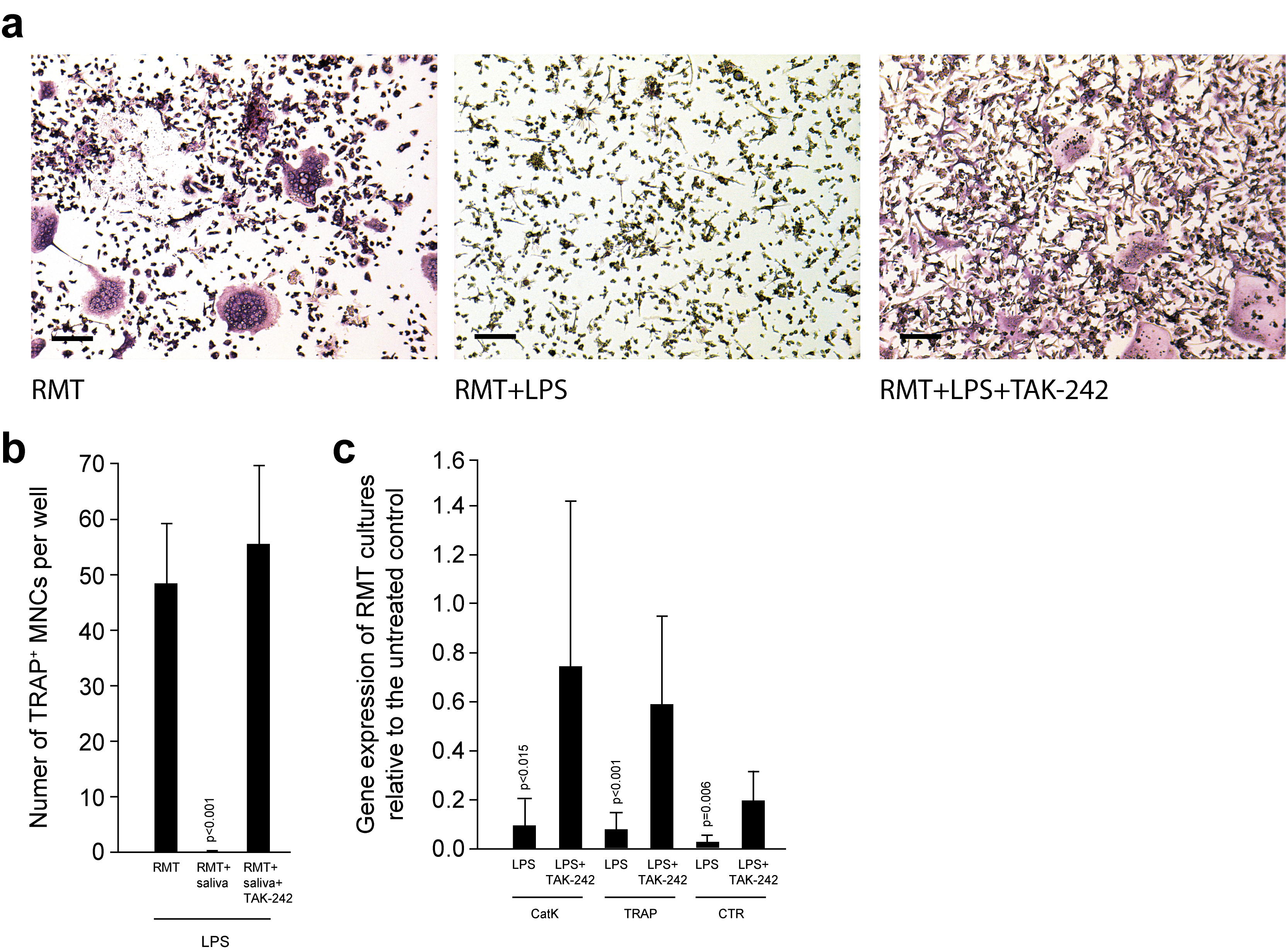

Supplement: Supplementary file 2 — High Resolution Image (TIFF 15216 kb) [file 784_2016_2041_MOESM1_ESM.tif]

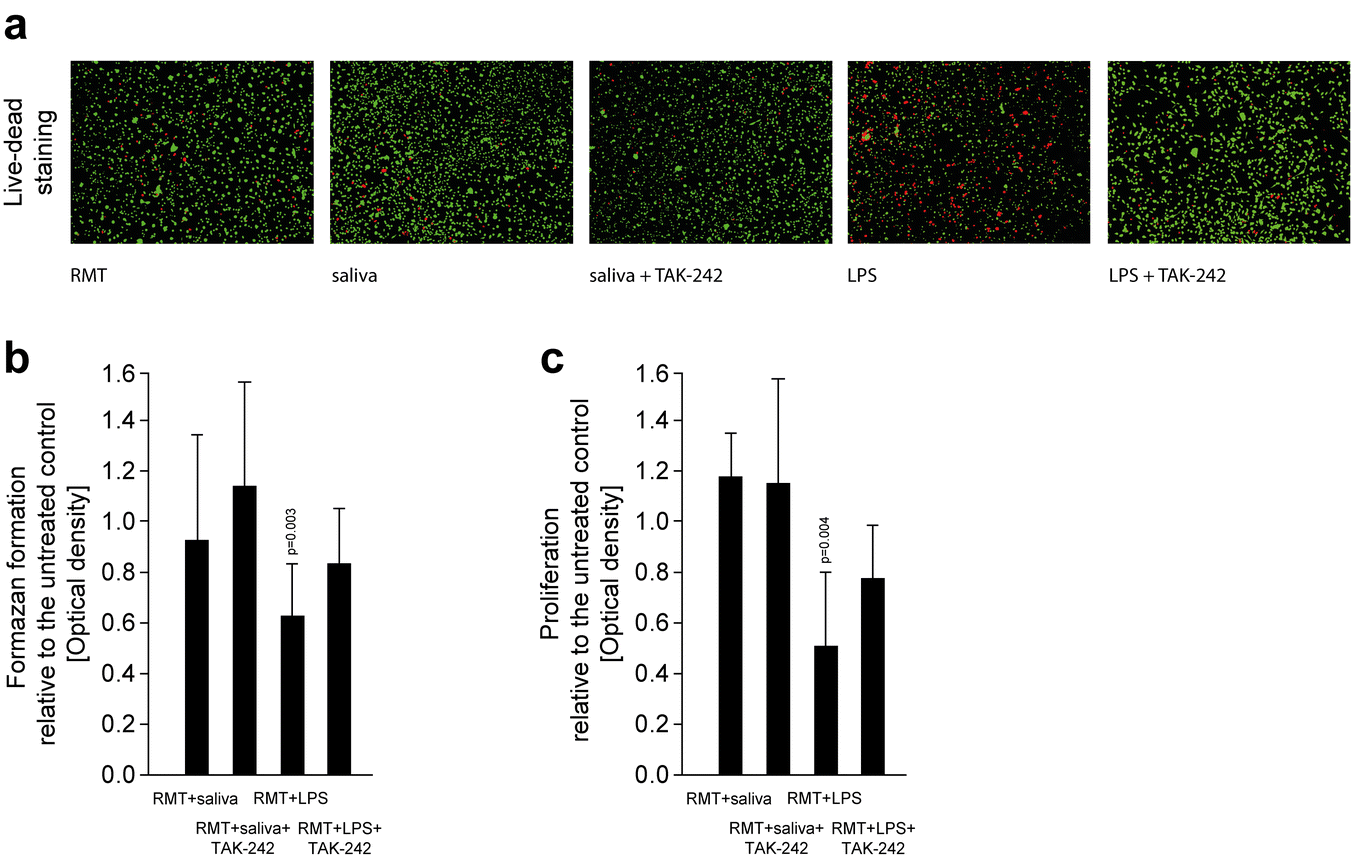

Supplement: Supplementary file 3 — Cell viability and proliferation in the presence of saliva, LPS and TAK-242. Osteoclast-like cells exposed to 10 μg/ml LPS showed significantly reduced viability and proliferation. For Live-dead staining, bone marrow cells were colored in green, indicating that the cells are viable (10-fold magnification) (a). The viability measures were determined via formazan formation assay (b). DNA incorporation of 5-Bromo-2´-Deoxyuridine (BrdU) Labeling and Detection Protocol was used to measure cell proliferation (c). Data were normalized to expression levels of RMT cultures. Bars represent the mean ± standard deviation of in total five experiments. Not indicated are p values>0.1 (GIF 198 kb) [file 784_2016_2041_Fig9_ESM.gif]

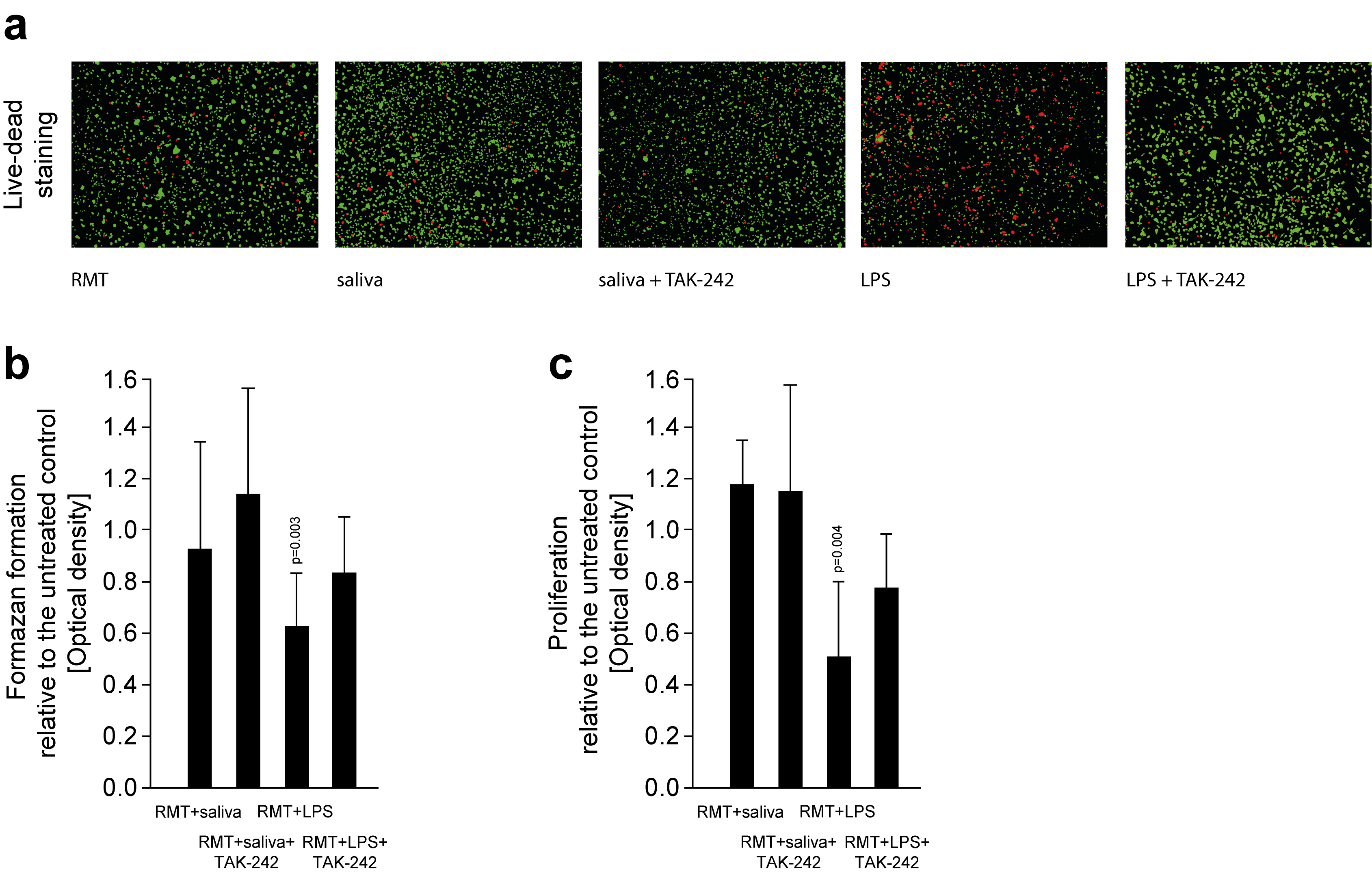

Supplement: Supplementary file 4 — High Resolution Image (TIFF 3689 kb) [file 784_2016_2041_MOESM2_ESM.tif]
